# Naturally occurring capsid protein variants L1 of human papillomavirus genotype 16 in Morocco

**DOI:** 10.6026/97320630013241

**Published:** 2017-08-31

**Authors:** Aissam El-Aliani, My Abdelaziz El Alaoui, Imane Chaoui, My Mustapha Ennaji, Mohammed Attaleb, Mohammed El Mzibri

**Affiliations:** 1 Unit of Biology and Medical Research, National Centre Natuional de l´Energie, des Sciences et des techniques Nucléaires. Morocco; 2 Laboratory of Virology Microbiology, Quality, Biotechnologies/Eco-Toxicology and Biodiversity (LVMQB/ETB), Faculté des Sciences et Techniques Mohammedia, Morocco; 3 National Center for Scientific and Technical Research CNRST, Morocco

**Keywords:** HPV 16, L1 protein, genetic variation, in silico prediction, vaccine

## Abstract

HPV L1 protein is a corner stone in HPV structure, it's involved in the formation of the viral capsid; widely used as a systematic material and considered as the main component in vaccines development and production. The present study aims to characterize genetic variation of L1 gene of HPV 16 specimens and to evaluate in silico the impact of major variants on the epitope change affecting its conformational structure. A fragment of L1 gene from 35 HPV 16 confirmed specimens were amplified by PCR and sequenced. Overall, five amino acids residues changes were reported: T390P in 16 specimens, M425I and M431I in 2 cases, insertion of Serine at 460 and aspartic acid deletion at position 477 in all analyzed cases. The 3D generated model showed that T389P amino acid substitution is located in the H-I loop; the two substitutions M424I and M430I are both located in the H2 helice. The Serine insertion and aspartic acid deletion are located in the H4 helice and B-C loop, respectively. Superimposition of sequences' structures showed that they share a very similar conformation highlighting that the reported amino acids variations don't affect the structure of the L1 protein. However T389P, located in the H-I loop identified as an immunogenetic region of L1 capsid, was reported in 51.4% of cases could interact with vaccines induced monoclonal antibodies suggesting a potential impact on the efficacy of available anti-HPV vaccines.

## Background

Worldwide, Cervical cancer is the fourth most common cancer in women, with an estimated 528,000 new cases in 2012, and more than 85% of the global burden occurs in developing countries, where it accounts for 13% of all female cancer [1]. There's evidence that persistent infection with high risk Human papillomavirus (HPV) is the main etiological factor in the development of cervical cancer [2]. Of these high-risk types, HPV-16 and HPV-18 are responsible for about 70% of cervical cancers [3]. Human papillomavirus (HPV) genomes are circular dsDNA characterized by eight open reading frames (ORFs), which are all transcribed from the same DNA strand and orientation, and yield two classes of proteins which are classified as non-structural regulatory proteins (E1-E7) and structural proteins L1 and L2 based on their temporal expression. Infectious HPV is primarily composed of 72 pentameric capsomeres of the L1 protein arranged in a T = 7-icosahedral capsid, the capsomeres are associated with 12 or more copies of the L2 protein [4]. The intrinsic capacity of L1 proteins to assemble into empty capsid-like structures has been used to develop virus like particles (VLPs) largely used in the induction of protective immunity in animal models [5] and the development of prophylactic vaccines for HPV infection [6,7,8]. Accordingly, two prophylactic vaccines; Cervarix (GSK) and Gardasil (Merck); based on the L1 proteins of HPV16 and HPV18, have been introduced into the immunization schedule in many developed and some developing countries [9].

On the surface of the pentamers, specific loops structures of the L1 protein contain type specific epitopes [10] and the vaccineinduced type-specific protection is likely mediated by neutralizing antibodies targeting L1 surface-exposed loops. Studies with monoclonal antibodies suggest epitopes composed of FG and HI loops are immunodominant for HPV 16 [11,12] whereas BC, DE, and HI loops are important for neutralization of HPV 6 and 11 [13]. Polymorphism within these loops is likely to result in the generation of neutralizing antibodies of different binding affinities due to the presence of different HPV types displaying distinct features on their surfaces [14]. Given that the prevalence of cervical cancer varies in different regions and countries, a number of studies have addressed the possible association of E6 based HPV16 variant status with different risks for progression to malignancy and suggested that HPV variants can influence the viral persistence and development of cervical cancer [15,16, 17,18]. Naturally occurring intratypic molecular variants of HPV-16 are defined as isolates with primary DNA sequence differences that total no more than 2% of the L1 open reading frame (ORF) of the prototype sequence [19] and are known to occur and have been shown to be specific or more prevalent in certain parts of the world [20].

Previous studies have reported that variations in L1 gene can affect the viral assembly affecting the protein structure or conformation and leading to altered biological functions with clinical significance, including the immunological recognition by the host [21,22]. On the other hand, L1 intratypic HPV variants can restrict the immune response by escaping consensus B- and T-cell epitopes of the available vaccines. These variants may also provide some new epitopes for targeting a particular geographical population, which may not be presented by these available vaccines [23]. In Morocco, as it is the case in the other North African countries, cervical cancer is the second most common cancer among women and its incidence is the highest in this region with an age standardized incidence rate (ASR) of 13.5 per 100 000 women [24]. In our previous HPV monitoring studies, we have shown that the two most prevalent high-risk HPV types among women, before and at the time of introduction of HPV vaccination in Morocco, were HPV16 and HPV18 [25,26, 27,28] and we have analyzed the intratypic variation of HPV16 based on the naturally occurring sequence variations of E6 and E7 genes, to have a global picture on the HPV16 variants circulating in Morocco [17,18]. However, to our best knowledge, there's no study giving information on the L1 variants of HPV16 in Morocco. Thus, the present study was planned to characterize genetic variation of L1 gene in a sample of Moroccan women with cervical cancer to identify the L1 HPV16 variants circulating in Morocco and to evaluate in silico the impact of major variants of L1 on the epitope change affecting the conformational immune reactive epitope regions within HPV16 genotypes.

## Methodology

### Clinical Specimens

DNAs from 35HPV16 positive cervical cancer samples were available from our laboratory DNA bank [17,18]. Overall, 36 cases (90%) were diagnosed at advanced stages (IIB and IIIB), whereas 4 patients (10%) was admitted at an earlier stage (IB). Pathological analysis revealed that all cases were squamous cell carcinoma (SCC) and only one case was a well differentiated adenocarcinoma. The ethic committee of Pasteur Institute in Morocco approved the study and written informed consent was obtained from each study subject.

### Variant analysis of L1 gene by PCR and direct sequencing

A fragment of 450 bp of L1 gene was amplified using MY09/MY11 consensus primers (MY09: 5'- GCMCAGGGWCATAAYAATGG-3'; MY11: 5'- CGTCCMARRGGA WACTGA-3'). PCR amplification was performed in a 25 μl volume containing 1,5mM MgCl2, 100 μM each dNTP, 0,2 μM forward and reverse primers, 100 ng genomic DNA and 0,25 U gold Taq DNA polymerase (Applied Biosystems, USA) in 1x PCR buffer. The amplification mixtures were first denatured at 94°C for 7 min. Then, thirty-five cycles of PCR were performed with denaturation at 94°C for 1 min, primer annealing for 1 min at 55°C and primer extension for 1 min at 72°C. At the end of the last cycle, the mixtures were incubated at 72°C for 7 min. For every reaction, a positive control, using DNA extracted from SiHa, an HPV16 positive cell line, and a negative control, without template DNA, was included. PCR products were tested on an ethidium bromide stained 2% agarose gel.

The ExoSaP ITR clean up system (USB, USA) was used to purify positive PCR products. Sequencing of purified PCR products was performed with BigDye Terminator v3.1 Cycle Sequencing Kit (Applied Biosystems). Sequencing reaction was performed in a final volume of 10μl containing 1μl of Big Dye v.3.1, 10 pmol of forward primer and 2μl of purified PCR product. The mixture was incubated at 96°C for 1min and 25 cycles were performed: denaturation at 96°C for 10s, primer annealing at 50°C for 5s and extension at 60°C for 4 min. The reaction was set to 30μl. To eliminate the excess of labeled ddNTPs, sequencing reaction products were purified using sephadex G- 50 gel-exclusion chromatography (GE Healthcare Life Sciences). Direct sequencing of amplified PCR products was performed on an ABI 3130xL Genetic Analyzer (Applied Biosystems).

### Sequences alignment

The compiled nucleotide sequences were aligned using ClustalW2 software [http://www.ebi.ac.uk/clustalw/] and the protein sequences were aligned using MUSCLE stands (MUltiple Sequence Comparison by Log-Expectation) [29]. SeqLogo was used to generate sequences logos from amino acid sequence alignment and evaluate the sequence variability and represent informations concerning consensus sequence [30].

### Phylogeny construction

The alignment was carried out using MUSCL and ClustalX2. Phylogenetic analysis was performed with the partial L1 sequences, which were constructed by the Maximum likelihood method and the Kimura 2-Parameter model by MEGA6, the Bootsrap proportions were calculated with 1000 replicates. The phylogenic tree has been evaluated by Shimodaira-Hasegawa-test and K-test. The thirty-five partial sequences of HPV16 variants were aligned with the prototype (GenBank: K02718.1) and other variants, As (GenBank: AF534061; AB889492), Af-1 (GenBank: AF472508; HQ644238), AA (GenBank: AF402678), and AA1 (GenBank: HQ644247) and E (GenBenk: NC_001526.2).

### 3D prediction of partial L1protein

The secondary structure of HPV16 L1 protein sequences was predicted using Swiss Model Server (http://swissmodel.expasy.org). Predicted 3D structures of HPV16 L1 were obtained using PHYRE2 Server [31], the alignment was done by the crystal structure of L1 protein of human papillomavirus 16 which was retrieved from Protein Data Bank (PDB) with PDB ID: 1dzl, the diffraction structure of L1 HPV protein had a resolution of 3.5 Å, R free value of 0.290 and R value of 0.280. PROCHECK server was used to evaluate the stereo chemical quality of protein models and to analyze residueby- residue geometry and the overall structure geometry [32]. The predicted partial protein structures of L1 HPV16, which present the amino acids variations, were generated, visualized and analyzed on PyMOL program [33].

## Results

Results from multiple sequence alignment of the 35 samples with the L1 reference sequence (ID: K02718.1) are reported in Table 1. Overall, 11 patterns have been reported. A total, of 17 single nucleotide changes have been reported overall. Among them, 5 were non-synonymous amino acids variations including A6693C observed in 18 cases, G6800A in 2 cases, G6818A in 2 cases. Of particular interest, ATC insertion at position 6901 and deletion of GAT at position 6950 were common to all analyzed samples.

Sequence analysis of a partial L1HPV nucleotide sequences' alignment has shown that 37.1% of the HPV16 variants identified were in the African 1, European and Asia lineage and 51.5% HPV 16 variant were in the African 2 lineage, while this tree shown one group of 11.4% variant which represent a low degree of evolutionary change compared with other variants (X lineage).

The thirty-five sequences were aligned using the MUltiple Sequence Comparison by Log- Expectation software (MUSCLE). SeqLogo uses this alignment to generate conserved sequences, the sequence conservation is indicated by the height of residue logos (bits). Overall, all sequences are presented with high residue logos level which means that all our sequences are conserved, except in two regions, H-I loop and B-C loop. In the first region the proline and threonine residues at position 390, are presented in equal proportion in our samples while in the second region all the specimens are characterized by the absence of aspartic acid at position 477. In Helice 2 region, the two mutations Met/Ile at position 425 and Met/Ile at position 431 are not significant as compared to the reference sequence. However, the insertion of serine at pos

Table 2 summarizes patterns associated with amino acids variation in the protein sequence. Among the 12 patterns obtained in our samples, only 3 gave modification in the primary protein structure. The first pattern, reported in 17 cases, is different from the L1 reference sequence by the ATC insertion at position 6901/6902 and the deletion of GAT at position 6950. The second pattern, reported in 16 cases, is characterized by the Thr389Pro change. In the third pattern, reported in only 2 cases, all non-synonymous mutations were reported (Thr389Pro, Met424Ile, Met430Ile, ATC insertion at position 6901/6902 and the deletion of GAT at position 6950).

The crystal structure of the HPV16 L1 protein was obtained from the RCSB Protein Data Bank (PDB id: 1dzl) and was used as a template to model the 4 HPV16 L1 patterns, including the reference sequence of L1 HPV protein (K02718.1). The quality of generated prototype model was evaluated using PROCHECK statistics and the obtained results are reported in the Ramachandran plot. Overall, 276 amino acids (AA) (71.2%) are located in the "Most favored regions" [A, B, L], 103 AA (26.5%) are located in the "Additional allowed regions" [a, b, c, l, p], 9 AA (2.3%) are located in "Generously allowed regions" [-a, -b, -l, -p] and no AA (0.0%) is located in the "Disallowed regions".

**Figure 3:**
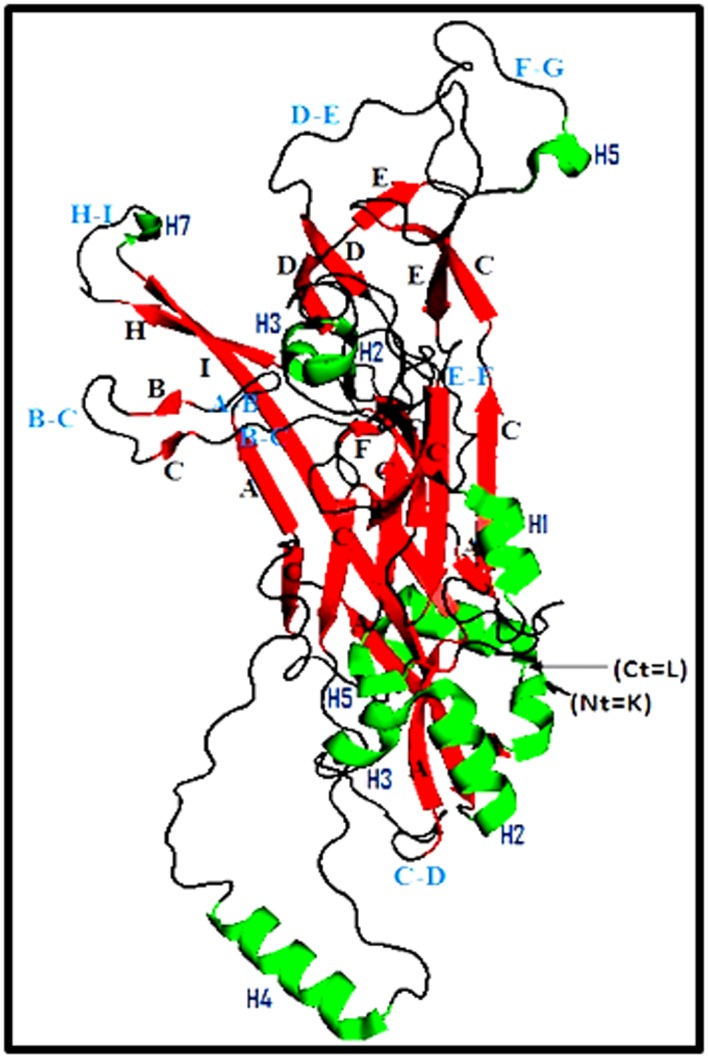
Carton structure of the major capsid protein (L1) of HPV16. (K02718.1). K: Lisyne, L: Leucine, Helix in green, Sheets in red, Loops in black), Ct: C-terminal amino acid, Nt: N-terminal amino acid.

The 3D L1 protein generated by PyMOL, representing an individual L1 protein molecule resulted from the expression of HPV16 L1-ORF, is reported in Figure 3. This structure showed the presence of 12 helices, 25 sheets and 26 loops. DE and FG loops known to interact with the L2 protein and producing conformational neutralizing antibodies. HI- and BC-loops variable regions are supposed to be involved in the interaction with the trans-regulatory protein E2.

**Figure 4:**
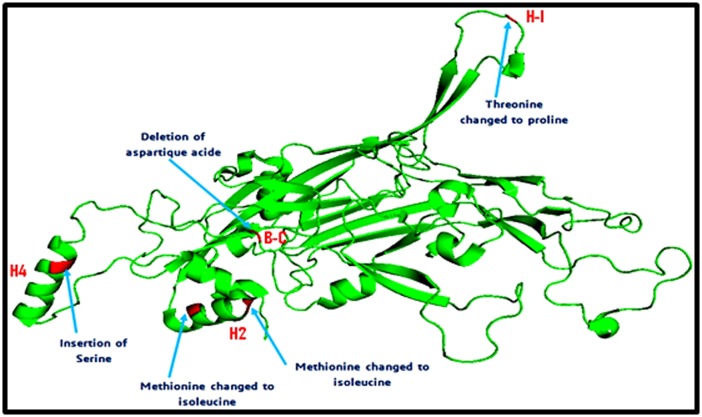
3D structure of L1 HPV16 protein showing the positions of the non-synonymous amino acids variation. Threonine changed to Proline, located in the H1 loop. Methionine in the position 424 was changed to Isoleucine and Methionine in position 430 was changed to Isoleucine are both located in the H2 helice. The serine insertion and aspartic acid deletion are located in the H4 helice and B-C loop, respectively.

Figure 4 illustrates the positions of the five non-synonymous mutations on the 3D structure of the HPV16 (L1) prototype. T389P amino acid substitution is located in the H-I loop; the two substitutions M424I and M430I are both located in the H2 helice. The Serine insertion at position 458/459 and aspartic acid deletion at position 475 are located in the H4 helice and B-C loop respectively.

**Figure 5:**
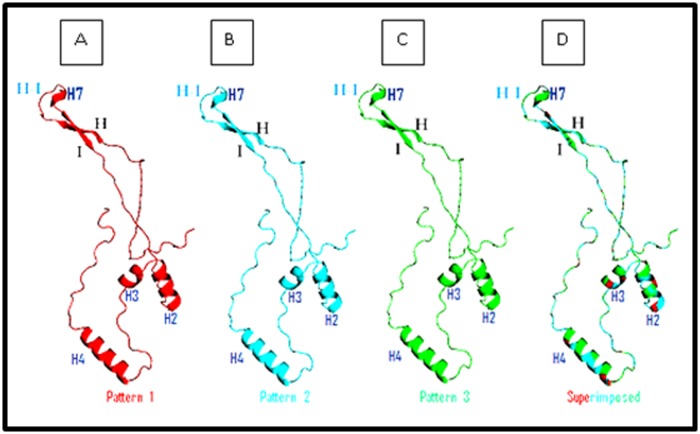
3D carton structure of HPV16 L1 homology models generated and visualized by PyMol. A: 3D sequence representation of the L1 fragments containing the insertion of Serine (460) and deletion of aspartic acid (477). B: 3D sequence representation of the L1 fragments containing the insertion of Serine (460), deletion of aspartic acid (477) and Thr/Pro substitution (390). C: 3D sequence representation of the L1 fragments containing the insertion of Serine (460), deletion of aspartic acid (477), Thr/Pro (390), Met/Iso (425) and Met/Iso (431) substitutions. D: Superimposition of both 3D structures with the prototype model showing that the structures share a very similar conformation.

The 3D carton structure of the L1 prototype and the three obtained structures are illustrated in Figure 5. The 4 generated models have been well designed with a score 1 obtained by Global Model Quality Estimation (QMEAN6 score). Superimposition of sequences' structures showed that they share a very similar conformation highlighting that the reported amino acids variations don't affect the structure of the L1 protein.

## Discussion

HPV16 and 18 L1 virus-like particles (VLP) are largely employed to develop prophylactic vaccines for the prevention of HPV infection. Currently, two prophylactic vaccines, Cervarix (GSK) and Gardasil (Merck), have been widely implemented in many countries. The efficacy of these vaccines, targeting highrisk HPV-16 and -18 alone, or additional HPV types, is still discussed, depending on the VLPs used to produce the vaccine and the HPV variants present in the targeted populations. Indeed, it's widely accepted that HPV variants have been shown to differ in geographic origins affecting the lesion progression and the vaccine protection [34,35,36].

In Morocco, prophylactic HPV vaccines were introduced in 2012 and are still with limited utilization. Thus, it's of a great interest to assess HPV16 and 18 L1 genetic diversities, at the introduction of vaccination, as a baseline, for a potential follow-up of the vaccination efficacy in the future. Previous studies conducted in Morocco have highlighted a high genetic diversity of HPV16 and the presence of both European (E), African (Af) and North- American (NA-1) phylogenetic clusters [17,18]. However, and to our best knowledge, there's no study giving information on the L1 variants of HPV16 in Morocco that could affect the efficacy of available vaccines. In this preliminary study, we have evaluated the HPV 16 L1 genetic diversity of 35 HPV 16 specimens previously isolated from cervical cancer patients in Morocco before the introduction of HPV vaccines. Genomic analysis of partial L1 sequence of HPV type 16 in these samples showed the presence of 17 nucleotide changes. Among them, five were non-synonymous; A/C (6694), G/A (6801), G/A (6819), Ins ATG (6903) and Δ GAT (6950).

Overall, 12 silent mutations were found in our specimens and most of them were already reported in many studies from India [37] Nederland [36] and Brazil [38]. Of particular interest, all the isolates analyzed differ from the reference sequence by the insertion of an ATC codon at position 6903 and deletion of GAT at positions 6951/2/3. Those mutations can represent a molecular signature of HPV circulating in Morocco. These two mutations were also reported in 100% of cases in India [37] and Brazil [38].

Partial sequence of L1 gene was used to have a phylogenetic analysis of HPV16 circulating in Morocco. Two different groups belonging to the African 1 and 2 lineages prevail, with 37.1% and 51.5% respectively. These results are in agreement with our previously reported data on the same isolates using E6 and E7 genes. Indeed, DNA sequencing of E6 and E7 genes highlighted that the predominance of HPV16 African variants and the majority of isolates belong to the African 1 and 2 lineages [17].

The computer modeling of L1 protein was generated for each pattern to assess the impact on the obtained non-synonymous mutations on the structure of the major capsid protein and therefore its potential interaction to available vaccines. In the L1 protein, the external loop regions: DE loop (AA 110-153), FG loop (AA 262-291), EF loop (AA 160-189), DC loop (AA 50- 69) and the H-I loop (AA 348-396) have been characterized as being antigenic [39]. Indeed, Carter *et al*. have shown that in human immune response, antibodies bind to regions DE, EF, FG and H-I loop, making these regions of a high interest for HPV VLP recognition [40]. Moreover, DE and FG loops interact with the proline-rich regions of the L2 whereas a cysteine residue within the EF loop is crucial for the formation of inter-capsomeric L1-L1 disulphide bonds [41].

Of particular interest, fine-mapping of the epitope footprint showed that the five non-synonymous changes of amino acids residues, including the ATC insertion and GAT deletion, are localized in the H2 and H4 helices and H-I and B-C loop regions T389P amino acid substitution is located in the H-1 loop, identified as an immunogenetic region of L1 capsid and believed to contribute towards cross-neutralising antibody [41]. M424I and M430I amino acid substitutions are located in the H4 helice whereas the Ser insertion is located in the H2 helice. Both structural elements H2 and H4 helices are near the C-terminal end of L1 and are important for the assembly of papillomaviruses into particles. Moreover, H2 region, in association with H3, is essential for L1 folding and pentamer formation, whereas the H4 region is indispensable for the assembly of both the virus particle and also T1 and T7 virus-like particle [6]. D475 deletion is located in the BC loop. BC loop contains lysine residues that can facilitate binding to heparin sulfate proteoglycans, the initial step required for successful HPV infection [41].

There's evidence that among the 5 non-synonymous mutations obtained in this study, 4 don't affect the immunogenic site of L1 and therefore don't interfere with the binding of monoclonal antibodies targeting HPV 16 and only the T389P amino acid substitution present in 51.4% of cases was associated with potential interaction to monoclonal antibodies induced vaccines. On the other hand, generated 3D carton structure of HPV16 L1 homology models, harboring the 5 non-synonymous mutations, clearly showed that the L1 structure rests unchanged suggesting that these mutations don't affect the overall structure of L1 protein.

The present study is very informative and give for the first time data on genetic diversity on HPV16 L1 gene in a Moroccan population and adress a prediction of potential reaction to available anti-HPV vaccines. However, the main limitation was the small number of HPV 16 DNA, which may not reflect the real situation in Morocco and give an exhaustive picture on L1 gene diversity and the mutational status highlighting mutations that could modify the L1 protein structure and consequently affect the neutralization epitopes.

## Conclusion

The present study gives evidence on the genetic diversity of HPV 16 L1 gene without any impact on the structure of the L1 protein. The study highlights the presence of T390P mutation, located in the H-I loop and could interact with vaccines induced monoclonal antibodies suggesting a potential impact of this mutation on the efficacy of available anti-HPV vaccines. Other studies are needed on large samples and by sequencing the entire HPV 16 L1 gene to predict the efficacy of HPV16 targeted vaccines and the success of the vaccination strategy in Morocco.

## Figures and Tables

**Table 1:** data analysis of L1 HPV sequences, represent nucleotide position, mutation and altered amino acid.

		6584	6591	6593	6634	6619	6800	6818	6833	6853	6863	6876	6887	6902	6923	6950	6968	6971
K02718.1		A	C	C	A	G	G	G	G	C	C	A	T	-	A	GAT	C	T
	N																	
Patterns	5	c	t	t		a				T				Ins<ATC>		Del	t	
	2	c	t	t										Ins<ATC>		Del		
	4		t	t										Ins<ATC>		Del		
	2	c	t	t										Ins<ATC>	g	Del		
	3		t	t					a					Ins<ATC>		Del		
	1		t	t		a				t				Ins<ATC>		Del	t	
	7	c	t	t	C	a				t	t			Ins<ATC>		Del	t	
	5		t	t	C	a				t	t			Ins<ATC>		Del	t	
	3		t	t	C	a				t	t	c		Ins<ATC>		Del	t	a
	1		t	t	C	a				t	t	c		Ins<ATC>		Del	t	
	2	c	t	t	C	a	A	A		t	t		g	Ins<ATC>		Del	t	
Original codon		GCA	GGC	CAC	ACT	AAG	ATG	ATG	TTG	CTA	CCC	ACA	ACT	-	AAA	GAT	TAC	ACT
Altered codon		GCC	GGT	CAT	CCT	AAA	ATA	ATA	TTA	TTA	CCT	ACC	ACG	ATC	AAG	Del	TAT	ACA
Original AA		A	G	H	T	K	M	M	L	L	P	T	T	-	K	D	Y	T
Altered AA		A	G	H	P	K	I	I	L	L	P	T	T	S	K	-	Y	T
Characteristic of AA					Polar/non polar		Non polar/ non polar	Non polar/ non polar						Polar		Polar		
		AA position					390		425	431						460		477		
Protein position					H-I loop		H2	H2						H4		B-C loop		

AA: amino acid, Ins: insertion, Del: deletion, A: alanine, T: threonine, P: proline, M: methionine, I: isoleucine, S: serine, D: aspartic acid. L: leucine, K: lysine, Y: tyrosine. H: helice																		

**Table 2:** Data analysis of four sequences presenting polymorphism in five amino acid residues

		6694	6800	6818	6902	6950
K02718.1		A	G	G	-	GAT
Patterns	N					
	17	A	G	G	Ins<ATC>	Del
	16	C			Ins<ATC>	Del
	2	C	A	A	Ins<ATC>	Del
Original codon		ACT	ATG	ATG	***	GAT
Altered codon		CCT	ATA	ATA	ATC	Del
Original AA		T	M	M	-	D
Altered AA		P	I	I	S	-
AA position		390	425	431	460	477
Protein position		H-I loop	H2	H2	H4	B-C loop

**Figure 1:**
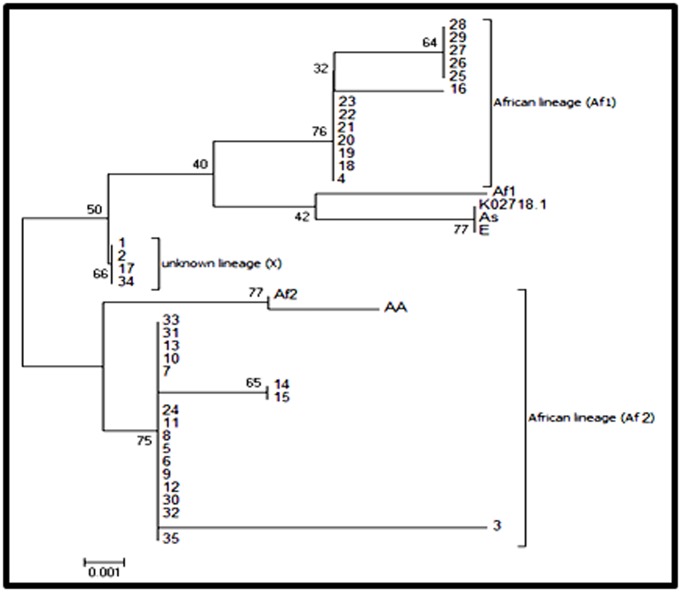
Phylogenetic tree of the Moroccan HPV16 variants. Phylogenetic study was constructed based on the partial L1 nucleotide sequence alignment of thirty-five sequences, which were constructed by the Maximum likelihood method and the Kimura 2-Parameter model by MEGA 6 package. Bootstrap proportions were calculated with 1000 replicates. Our study sequences are shown in Arabic numbers; others are shown with abbreviations, African lineage (Af), European prototype (Ep). European variant (E), Asia lineage (As), Asian American lineage (AA) and the prototype (K02718.1).

**Figure 2:**
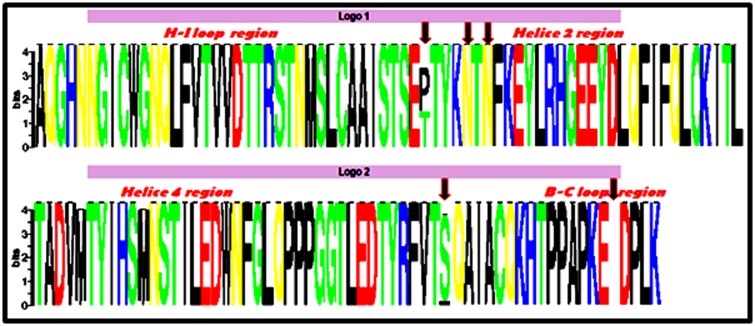
Variability of amino acid residues using SeqLogo. The twenty-three sequences have been used to estimate amino acids residue variation using SeqLogo. Sequence conservation is represented by the height of residue logos “indicated in bits”, and the arrow indicates the change in amino acids residues.
